# Low rates of antibiotic resistance and infectious mortality in a cohort of high-risk hematology patients: A single center, retrospective analysis of blood stream infection

**DOI:** 10.1371/journal.pone.0178059

**Published:** 2017-05-19

**Authors:** Jason R. Conn, Elizabeth M. Catchpoole, Naomi Runnegar, Sally J. Mapp, Kate A. Markey

**Affiliations:** 1 Division of Cancer Services, Princess Alexandra Hospital, Brisbane, Queensland, Australia; 2 Pathology Queensland, Royal Brisbane and Women’s Hospital, Brisbane, Queensland, Australia; 3 The University of Queensland, School of Medicine, Brisbane, Queensland, Australia; 4 Infection Management Services, Princess Alexandra Hospital, Brisbane, Queensland, Australia; 5 Haematology and Bone Marrow Transplantation Unit, Royal Brisbane and Women’s Hospital, Brisbane, Queensland, Australia; 6 QIMR Berghofer Medical Research Institute, Brisbane, Queensland, Australia; 7 Australian Infectious Diseases Research Centre, University of Queensland, Brisbane, Queensland, Australia; GERMANY

## Abstract

Febrile neutropenia (FN) is a medical emergency and can represent a life-threatening complication for hematology patients treated with intensive chemotherapy regimens. In clinical practice, the diagnostic yield of blood cultures and other investigations which aim to identify a causative organism or site of infection is low. We have retrospectively examined all blood cultures collected in a “real world” cohort of patients receiving chemotherapy for acute leukemia and patients with aggressive lymphoma treated with Hyper-CVAD/MTX-cytarabine, at a single tertiary center over a five-year period. In this cohort, the 30-day mortality following confirmed blood stream infection (BSI) was 5.9%, which is lower than most reports in the recent literature. We compared the blood culture results of inpatients undergoing induction chemotherapy and outpatients presenting with fevers and found a significantly higher rate of proven BSI in the outpatient group. In all settings, gram-negative organisms were most common. The rate of resistance to first-line empiric antibiotics among pathogenic isolates was 11.6% in the whole cohort, independent of blood culture circumstances. There was a trend to higher resistance rates among inpatients undergoing induction chemotherapy compared to patients presenting to the emergency department (17.4% vs 7.5%) but this did not reach statistical significance. We also report low rates of ciprofloxacin resistance (5% of isolates), in a center where universal fluoroquinolone prophylaxis is not employed. Our low resistance and mortality rates support our current therapeutic strategies, however presence of resistant organisms across the spectrum of indications for BC collection highlights the importance of surveilling local patterns, escalating antimicrobial therapy in the deteriorating patient, and considering advanced techniques for the rapid identification of resistance in this patient population.

## Introduction

Patients undergoing therapy for hematological malignancy are at significant risk of life-threatening infection due to the immune-suppressive nature of their underlying disease, as well as chemotherapy-induced neutropenia [1, 2]. Febrile neutropenia (FN) is a medical emergency, leads to a large number of hospital admissions and contributes to morbidity and mortality in the hematology patient population [3–5]. Fever may represent the only hallmark of blood stream infection (BSI) in the neutropenic patient, with the usual focal symptoms and signs typically attenuated by the absence of functional innate immunity [6, 7]. Progression to septic shock and multi-organ failure carries a mortality of up to 40% when gram-negative organisms are implicated. FN also has a significant impact on morbidity and an indirect effect on mortality due to delays in chemotherapy administration and alterations in dosing [3, 8].

Given the potentially grave outcome of neutropenic sepsis, and the evidence supporting improved outcomes with rapid antibiotic therapy, current treatment protocols for the febrile hematology patient involve blood culture collection followed by immediate administration of broad-spectrum antibiotics [9]. Despite a diagnostic focus on bacteremia and concern about resulting clinical sepsis, microbiological confirmation of BSI only occurs in 15% to 35% of cases of FN, and reported in-hospital mortality ranges from 3 to 39% [3, 4, 9]. This wide range is attributable, in part, to the emerging challenge of multi drug-resistant organisms in some populations, particularly the gram-negative bacteria [10]. The frequency of BSI with multi-drug resistant organisms is as high as 35% in some centers, with mortality in these patients ranging from 35% to 80% [11, 12].

We performed a retrospective analysis of the highest-risk hematology patients in our center, aiming to establish the incidence of microbiologically proven BSI, define the causative organisms and establish rates of antibiotic resistance in our usual clinical practice. We hypothesized that the ambulatory patient group and the inpatients receiving induction chemotherapy would have differing rates of proven BSI, and that there would be higher rates of resistance to first-line antimicrobials in the hospitalized group.

Given our low mortality, the data highlight the safety of a fluoroquinolone-free prophylactic regimen (valaciclovir, trimethoprim/sulfamethoxazole and fluconazole or posaconazole) for patients undergoing treatment for high risk hematological malignancies in a setting with low background rates of gram-negative multi-resistant organisms. We confirm a low rate of resistance to broad spectrum beta-lactam antibiotics and an aminoglycoside when these are used empirically in combination, and failed to identify any difference in resistance to first-line antibiotics between in and outpatient groups, though there was a trend toward higher rates of resistance in the inpatients. Overall rates of blood culture positivity and resistance support the use of broad-spectrum antibiotics in new febrile neutropenic presentations, and confirm the importance of accurate knowledge of patient-specific risk of infection with resistant organisms in tailoring initial therapy and escalation strategies.

## Materials and methods

### Study population

Patients were treated at Princess Alexandra Hospital, a tertiary referral hospital in Brisbane, Australia, from January 2010 to December 2014. On the basis of exposure to chemotherapy regimens which almost universally induce profound neutropenia we included the following patients in our high-risk cohort: 1) those treated with induction and consolidation chemotherapy for acute leukemia [acute myeloid leukemia (AML), acute promyelocytic leukemia (APML), acute lymphoblastic leukemia (ALL)], or other disease as described in Table 1, and 2) patients with aggressive lymphoma treated with Hyper-CVAD/MTX-cytarabine chemotherapy (hereafter referred to as HCVAD), as this was the regimen of choice for high-risk disease in our institution. While autologous (but not allogeneic) transplants are performed at our center, numbers are small and therefore these patients were not included in the analysis. All treatment regimens were confirmed by multidisciplinary team consensus. Standard prophylaxis in this institution consisted of valaciclovir and trimethoprim/sulfamethoxazole. Ciprofloxacin was not in routine use as prophylaxis in our center for the study period, and is not in current use now. Posaconazole was used in the AML cohort as prophylaxis for fungal infection, and fluconazole in all other patients. Granulocyte-colony stimulating factor (G-CSF) was prescribed for all patients.

**Table 1:** High risk patient demographics, 2010–2014.

Characteristic	Acute Leukemia	High risk lymphoma
n	151	61
Number female (%)	70 (46.4%)	18 (29.5%)
Age (median +/- SD)	51 +/- 17.3	53 +/-15.5
Diagnosis, n (%)		
Acute myeloid leukemia	93 (62.4)	-
Acute promyelocytic leukemia	22 (14.7)	-
B-acute lymphoblastic leukemia	23 (15.4)	-
T- acute lymphoblastic leukemia	7 (4.6)	-
Other (acute leukemia group)^a^	6 (4.6)	-
Diffuse large B cell lymphoma (DLBCL)	-	12 (19.7)
Mantle cell lymphoma	-	10 (16.39)
Burkitt Lymphoma	-	10 (16.4)
DLBCL/Burkitt Intermediate	-	6 (9.84)
Plasmablastic lymphoma	-	5 (8.2)
Lymphoblastic lymphoma	-	4 (6.5)
Anaplastic large cell lymphoma	-	5 (8.2)
Other (high risk lymphoma group)^b^	-	9 (14.6)

^a^ “Other” patients in the acute leukemia group included 1 patient with refractory anemia with excess blasts-2 (RAEB-2) myelodysplastic syndrome who received induction chemotherapy, 2 patients each with blast-crisis chronic myeloid leukemia and acute erythroleukemia, one patient with blastic plasmacytoid dendritic cell neoplasm, and one patient with myelodysplastic syndrome/myeloproliferative neoplasm (MDS/MPN) overlap disease with leukaemia cutis.

^b^ “Other” patients in the high risk lymphoma group included one patient with each of the following conditions: B cell lymphoproliferative disorder unclassifiable, with leptomeningeal and bone marrow involvement, gamma-delta T cell lymphoma, cutaneous T cell lymphoma, DLBCL/cHL intermediate lymphoma, aggressive B-NHL not otherwise specified, undifferentiated haematological malignancy (CD43+, Bob-1+), NKT cell lymphoma, and 2 patients with AITL.

Patients were identified using an institutional leukemia and lymphoma patient database, which was cross-checked against the state-wide laboratory information system *(Auslab)*, and institutional chemotherapy prescribing software *(CHARM)*. Admissions data was available via *'The Viewer*' system used within Queensland Health, which collates admission and discharge summary data.

For all patients studied, the following data was collected: demographic information, specific diagnosis, date of diagnosis, treatment regimen, number of admissions to hospital (presenting complaint, length of stay, number of blood cultures drawn, number of blood cultures positive, absolute neutrophil count (ANC) on presentation). Neutropenia was defined as ANC ≤ 0.5 x 10^9^/L. Mortality data were collected and BSI-associated mortality was defined as any death within 30 days of laboratory confirmed BSI.

### Empiric antimicrobials

From 2012, the standard antimicrobial regimen consisted of piperacillin/tazobactam 4/0.5g, 6 hourly, and gentamicin 4-7mg/kg. The gentamicin is typically included for 1–2 initial doses, based on the historical presence of a small but significant number of piperacillin/tazobactam resistant gram-negative organisms. For 2010–2011, ticarcillin/clavulanic acid was the broad-spectrum beta-lactam of choice. Vancomycin was added if there were clinical concerns regarding potential central venous access device (CVAD) source or severe sepsis, but was not generally part of initial therapy.

### Blood culture definitions

Blood cultures were collected using standard clinical procedures, incubated and monitored for positivity using the BacT/Alert 3D system (bioMérieux, Marcy l’Etoile, France), and antimicrobial susceptibility testing was performed as per local laboratory protocols using Clinical and Laboratory Standards Institute standards [13], transitioning to European Committee on Antimicrobial Susceptibility Testing standards in 2011 [14]. Where a positive blood culture was returned, the specific organism and antimicrobial susceptibility profile were collected. An organism was considered resistant if it demonstrated resistance to our institution’s empiric antibiotic regimen for hematology patients. Coagulase-negative Staphylococci (CoNS), *Propionobacterium acnes*, and *Corynebacterium*, *Micrococcus*, *Microbacterium*, *Brevibacterium* and *Bacillus* species were isolated in this series, but were only considered to be of clinical significance if isolated in parallel from all lumens of a central venous line (CVL) and a peripheral blood culture; this occurred in one instance [12, 15]. A clinical blood culture ‘episode’ was defined as a group of blood cultures collected not more than 4 hours apart.

### Statistics

**Data were compiled using Microsoft Excel and statistical analysis was performed using GraphPad Prism using GraphPad Prism version 7.00 for Windows (GraphPad Software, La Jolla California USA,** www.graphpad.com**). Contingency analysis followed by Fishers exact test and Mann-Whitney non-parametric analyses were employed.Ethics approval.** Ethics approval was granted by the Human Research Ethics Committee of the Princess Alexandra Hospital (HREC 15/QPAH/484). Given the retrospective nature of the study, patients were not approached for individual consent. This was approved by the HREC.

## Results

### Patient characteristics, mortality and BSI rates

For the study period January 2010 to December 2014, a total of 212 patients (151 acute leukemia, 61 high-risk lymphoma) were identified and patient characteristics are summarized in Table 1. This is broadly representative of the cases treated in other hematology centers. All-cause mortality within 30 days of blood stream infection was documented in 9 patients (5.9%). Of the 7 occurring in the acute leukemia group, 3 occurred during AML induction, and 2 occurred in the setting of HCVAD therapy for B-ALL or CML in blast crisis. Two deaths occurred in the HCVAD cohort, both in patients receiving therapy for DLBCL. Organisms isolated were: *Clostridium septicum (2 cases)*, *Enterobacter cloacae*, *Pseudomonas aeruginosa*, *Stenotrophomonas maltophilia*, *Serratia marcescens*, *and Escherichia coli (extended-spectrum beta-lactamase (ESBL) producing)*.

**Fig 1:**
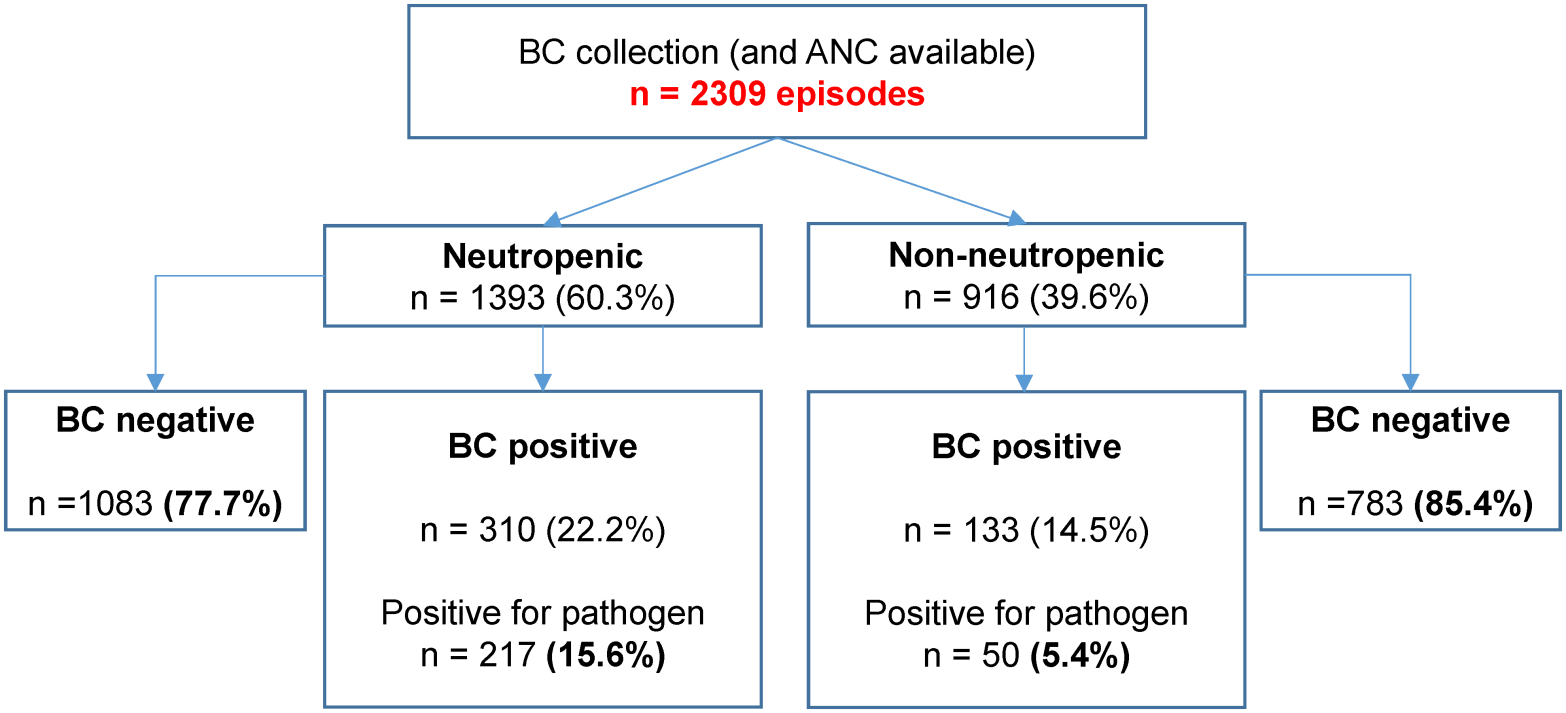
Blood culture episodes. Blood culture episodes are divided into neutropenic (ANC <0.5 x 10^9^/L) and non-neutropenic. Cultures are classified into negative and positive, and the positive results are sub-classified depending on whether they were positive for a contaminant or a pathogenic organism.

In the cohort as a whole, blood cultures were collected on 2324 occasions in the study period, and a corresponding neutrophil count was available in almost all instances (2309 occasions, 99.3%). As described in Fig 1, 60% of these BC were drawn when patients were neutropenic, and had a corresponding rate of proven BSI of 15.6% compared to 5.4% in the non-neutropenic BC episodes (p <0.0001; OR 3.9, 95% CI 2.6–6.00).

Among neutropenic patients, after discounting the contaminants isolated, organisms were predominantly gram-negative (76.2%), with *Escherichia coli*, *Enterobacter cloacae*, *Pseudomonas aeruginosa and Klebsiella pneumoniae* accounting for 90.4%. The remainder were: 19.9% gram-positive (the majority *Streptococcus* and *Staphylococcus* species), 3% anaerobes and a single episode where *Candida tropicalis* was isolated. There were no significant differences in the organism types isolated in neutropenic and non-neutropenic patients when a pathogenic organism was confirmed.

High-risk febrile patients typically receive initial antibiotics as per neutropenic fever guidelines, before the full blood count data is available from the laboratory. Therefore, both neutropenic and non-neutropenic hematology patients receive the same empiric antibiotics on initial presentation. In practical terms, it is therefore most important to consider the number of occasions where this therapy would be appropriate in light of the organisms ultimately isolated. Susceptibility to the combination of piperacillin/tazobactam (or ticarcillin/clavulanic acid prior to 2012) and gentamicin was confirmed in 88.4% of isolates. This rate did not differ among neutropenic and non-neutropenic patients (88% vs 90%, *p = 0*.*81)*. Of note, ciprofloxacin resistance was rare, only occurring in 5% of isolates. Again, this was not different when neutropenic and non-neutropenic patients were compared (5.2% vs. 6.3%. *p = 0*.*81)*.

In all patients, a total of 31 isolates demonstrated resistance to empiric antibiotics. Seventeen (54.8%) had acquired resistance or decreased susceptibility to first line antibiotics (5 penicillin-resistant *Streptococcus* species, 5 ESBL producing *Escherichia coli*, 3 methicillin resistant *Staphylococcus aureus*, 2 vancomycin resistant *Enterococcus faecium* (VRE) and 1 *Granulicatella adiacens*. Fourteen isolates (45.1%) were intrinsically resistant and included 6 episodes of non-vancomycin resistant *Enterococcus faecium*, 5 episodes of *Stenotrophomonas maltophilia* isolation, and 3 of *Enterobacter cloacae*. To clarify these results further, we next examined rates of proven BSI after febrile presentations to the emergency department and during the initial inpatient stay for induction chemotherapy.

### Blood stream infections during hospitalization for induction chemotherapy

**Fig 2:**
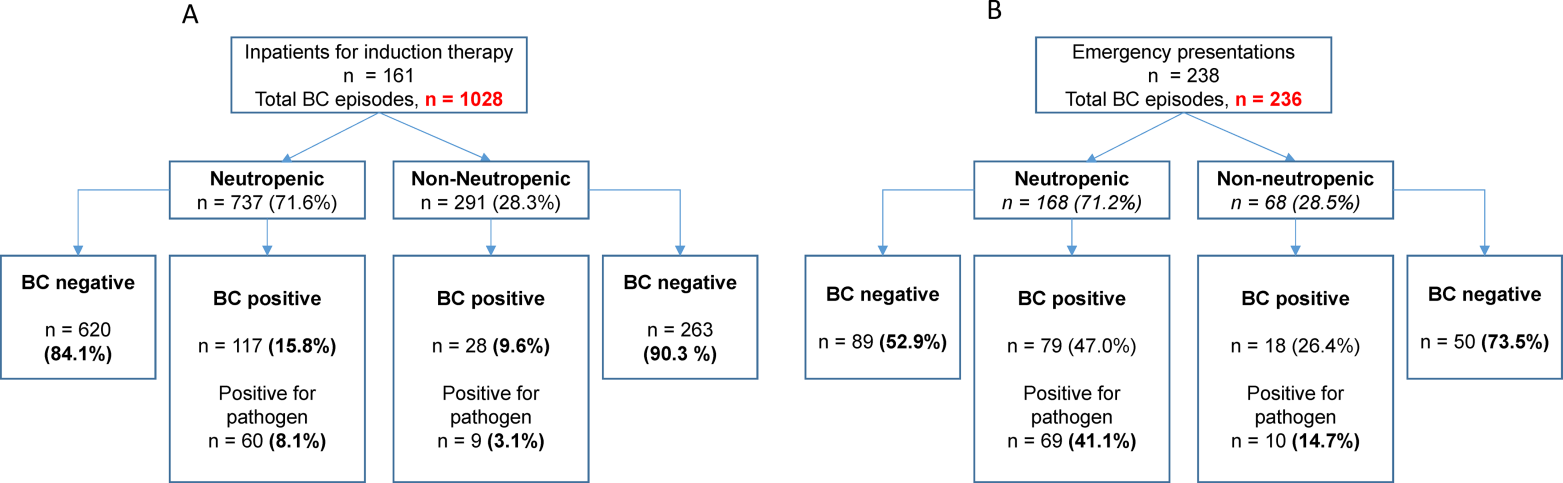
Blood culture episodes among inpatients receiving induction chemotherapy and outpatients presenting to the emergency department. A) Blood culture episodes among the 161 patients admitted for induction chemotherapy, divided as described in Fig 1. B) Blood culture episodes among patients presenting to the emergency department with a febrile episode.

Of the 212 patients, 161 were inpatients for induction chemotherapy (as summarized in Fig 2). We hypothesized that a different group of organisms and antimicrobial susceptibilities may be present in these patients compared to the ambulatory group. Inpatients are at high risk for nosocomial infections due to prolonged admissions associated with induction therapy and its potential complications [11, 16].

The AML group comprised the majority of the acute leukemia sub-group, accounting for 93 patients. A majority of these (62/93 patients; 66.6%) were treated with a “7+3” cytarabine and idarubicin regimen. Other induction therapies included high-dose cytarabine (HiDAC) + idarubicin (7.5%), fludarabine + high dose cytarabine + G-CSF (FLAG) +/- idarubicin (7.5%), ifosfamide + carboplatin + etoposide (ICE, 6.4%), and various clinical trial protocols (5.3%). Outside the AML patients, induction therapies included: HCVAD “A” cycles, the ALL-BFM 95 protocol, and Idarubicin, all-trans-retinoic acid and arsenic for patients treated for acute promyelocytic leukemia using the APML4 approach [17]. These are all intensive chemotherapy regimens that result in prolonged myelosuppression, even with the use of G-CSF to promote neutrophil recovery.

During the study period, 1028 discrete clinical episodes demanded clinical blood culture collection, most of which occurred in neutropenic patients (71.6%, Fig 2A). BSI was confirmed in 8.1% of cases when the patient was neutropenic and 10 episodes (3.1%) when the patient was non-neutropenic (p = 0.0022; OR 2.828, 95% CI 1.42–5.645). As in the all-comers patient group, gram-negative organisms represented the majority of these infections. Fungaemia was only confirmed in one patient (Table 2).

**Table 2:** Organisms isolated in the cohort, by ANC at time of collection.

	Neutropenic (ANC ≤0.5 x 10^9^/L)		Non-neutropenic (ANC >0.5 x 10^9^/L)	
Proven BSI (n)	310		133	
**Contaminant**	91	*(29%)*	83	*(62%)*
**Non-contaminant**	219	*(71%)*	50	*(38%)*
**Gram-negative**	161	*(74%)*	34	*(68%)*
*Escherichia coli*	48	*(30%)*	9	*(26%)*
*Enterobacter cloacae*	36	*(22%)*	2	*(6%)*
*Klebsiella species*	33	*(20%)*	4	*(12%)*
*Pseudomonas species*	31	*(19%)*	6	*(18%)*
*Acinetobacter species*	3	*(2%)*	4	*(12%)*
**Gram-positive**	42	*(19%)*	9	*(18%)*
*Streptococcus species*	20	(48%)	1	*(11%)*
*Enterococcus species*	10	(24%)	4	*(44%)*
*Staphylococcus aureus*	7	(17%)	3	*(33%)*
**Anaerobes**^a^	5	*(2%)*	4	*(8%)*
**Multipathogen**^b^	8	*(4%)*	3	*(6%)*
**Fungus**^c^	1	*(0*.*3%)*	0	-

^a^Anaerobes isolated were: *Bacteroides fragilis*, *Clostridium septicum*, *Egerthella lenta* and *Lactobacillus species*.

^b^ Multipathogen infections were comprised of: *Klebsiella oxytoca + Serratia liquifaciens*, *Klebsiella pneumoniae + Achromobacter xyloxidans*, *Klebsiella pneumonia + Stenotrophamonas maltiphilia*, *Eschericia coli + Enterobacter cloacae*, *Raoultella ornitholytica + Staphylococcus aureus*, *Klebsiella pneumoniae + Pseudomonas aeuruginosa*, *Streptococcus mitus and salivarius*, *Fusobacterium nucleatum + Streptococcus mitis*, *Klebsiella pneumoniae + Proteus vulgaris*, *and Eschericia coli + Klebsiella pneumoniae + Streptococcus vestibularis*.

^c^ *Candida albicans*

Organisms isolated among inpatients were susceptible to empiric antibiotics in 82.6% of cases which was lower than that seen in the cohort as a whole, but this did not reach statistical significance (*p = 0*.*22)*.

Positive blood cultures predominantly occurred at the time of the expected neutropenic nadir in this patient group though late positive cultures were observed when patients required a prolonged hospital stay. The mean duration of a patient’s first admission was 34 days.

### Blood culture results following emergency presentations in high-risk patients

Patients in the cohort had 238 discrete emergency presentations in which outpatients presented to hospital with fever (Fig 2B). There was no statistical difference between the leukemia and lymphoma groups with respect to their risk of presenting to hospital with fever during the course of their therapy (*p = 0*.*22)*. Ninety-seven patients had positive blood cultures upon presentation (40.75%), the majority of these occurring in patients who were neutropenic (79/97, 81.4%). For those patients who were neutropenic at presentation (mean ANC 0.036 +/- 0.079 x10^9^/L), 41.1% returned a positive culture for a pathogenic organism, compared with 14.7% in the non-neutropenic group (*p* = 0.002; OR 3.87, 95% CI 1.82–7.825).

Gram-negative bacteria were cultured in 82.3% of positive episodes; *Escherichia coli* was the most common of these organisms, isolated in 25.3% of cases. Only 11.4% of isolates were gram-positive species, with *Staphylococcus aureus* and *Streptococcus mitis* together comprising 66.7% of gram-positive infections. Co-infection was relatively uncommon, occurring in 6.3% of cases, involving predominantly gram-negative species (Table 3). Importantly, 92.5% of organisms isolated were susceptible to our local empiric antibiotic regimen. This was higher, but not statistically different to the rate seen in the inpatient cohort (p = 0.08), or the rate across the entire patient cohort for all clinical scenarios (p = 0.8).

**Table 3:** Organisms by inpatient for induction chemotherapy vs outpatient emergency presentation.

	Inpatient for induction		Emergency presentations		*p*
Total positive cultures	145		97		
**Contaminant**	76	*(52*.*4%)*	18	*(18*.*6%)*	
**Non-contaminant**	69	*(47*.*6%)*	79	*(81*.*4%)*	*<0*.*0001*
**Gram-negative**	44	*(63*.*8%)*	65	*(82*.*3%)*	*0*.*0147*
*Escherichia coli*	10	*(22*.*7%)*	20	*(30*.*8%)*	
*Enterobacter species*	6	*(13*.*6%)*	15	*(23*.*1%)*	
*Klebsiella species*	12	*(27*.*3%)*	15	*(23*.*1%)*	
*Pseudomonas species*	7	*(15*.*9%)*	13	*(20*.*0%)*	
*Acinetobacter species*	2	*(4*.*5%)*	-	-	
*Stenotrophamonas maltophilia*	3	*(6*.*8%)*	1	*(1*.*5%)*	
*Moraxella cattarhalis*	0	-	1	*(1*.*5%)*	
*Other*	4	*(9*.*1%)*	0	-	
**Gram-positive**	17	*(24*.*6%)*	8	*(10*.*1%)*	*0*.*0269*
*Streptococcus species*	6	(35.3%)	4	*(50*.*0%)*	
*Enterococcus species*	7	(41.2%)	1	*(12*.*5%)*	
*Staphylococcus aureus*	1	(5.9%)	3	*(37*.*5%)*	
*Other*	3	(17.6%)	-	-	
**Anaerobes**	3	*(4*.*3%)*	2	*(2*.*5%)*	*0*.*66*
**Multipathogen**	4	*(5*.*8%)*	5	*(6*.*3%)*	*>0*.*9999*
**Fungus**	1	*(0*.*7%)*	0	-	*0*.*4662*

### Role of blood cultures in patients with recurrent fevers

Controversy remains as to the clinical value in repeating blood culture collection in patients who are persistently febrile after the commencement of empiric antibiotics [18]. In general, the causes of ongoing fevers despite broad-spectrum antimicrobial therapy are wide-ranging, and include blood stream infection with resistant organisms, invasive fungal infection, drug-related and disease-related fevers [19]. In our cohort, 144 presentations lead to repeat blood cultures (60.8%) and these rarely led to an alteration in clinical management (8 cases, or 5.6%, over the five year study period). In the majority of these cases (6 of 8) the repeat cultures had been collected after the patients’ admission duration had exceeded two weeks; 5 of these episodes were associated with clinical signs of sepsis in a previously well patient who had remained in hospital to receive further chemotherapy. The positive culture was useful in the sixth long-staying patient who had persistent fevers despite broad antimicrobial therapy (with meropenem), however remained hemodynamically stable. For the remaining two cases across the study period, repeat BC were collected within 5 days of admission for persistent fever. One case returned a second pathogen requiring a change in antimicrobial therapy, and a positive BC was obtained in the other patient who had initially been culture negative. Reculture practice was similar among the neutropenic and non-neutropenic patients.

## Discussion

Our 30-day BSI-related mortality rate of 5.9% in high-risk hematology patients is amongst the lowest reported in the literature, although inconsistencies in reporting of mortality and variability in patient groups studied make comparisons to other centers challenging. Some studies of febrile neutropenia in the general cancer patient population have reported similar low mortality, but recent studies from individual Spanish and American centers have reported case-mortality of 12.1% and 16.8% for BSI among the hematology patient population [20–22]. A recent prospective Italian study of *Klebsiella pneumoniae* (KP) BSI in hematology oncology patients reported 21-day BSI-related mortality of 14.5% in their patients without carbapenem-resistant organisms, and a mortality of 52.2% in those with carbapenem resistant organisms isolated [23]. This is likely attributable to the high rates of antimicrobial resistance and thus, inadequate initial antibiotic therapy in these centers, as the authors report that even when carbapenem-sensitive KP was isolated, 22% of patients were inadequately treated by the broad-spectrum first-line antibiotics of choice in their center.

Our data demonstrate that we are likely to confirm a BSI in approximately one-third of cases when our ambulatory patients present to hospital febrile, with this rate rising to 40% if patients are neutropenic. Organisms isolated were predominantly gram-negative, which is consistent with recent global trends. However, contrary to worldwide patterns regarding antimicrobial resistance, in our center the organisms isolated remain relatively sensitive to our institutional empiric therapy of piperacillin/tazobactam (ticarcillin/clavulanic acid prior to 2012) and gentamicin and have very low rates of resistance to ciprofloxacin. In the group of patients studied here, exclusion of gentamicin would have meant that a further 13 ambulatory patients, and 15 episodes among the inpatients receiving induction chemotherapy would not have been treated with appropriate initial empiric therapy. This would escalate the resistance rates to 13.3% in the cohort as a whole, 24.1% in the ambulatory patients and 39.1% in the inpatient cohort. In this latter group, the increase is driven by the rates of *Enterobacter*, *Citrobacter and Acinetobacter* BSI. In our view, this justifies the continued inclusion of gentamicin in our institution, and is in keeping with current Australian Therapeutic Guidelines for empiric antimicrobials in neutropenic sepsis [24].

In our center, which is a large tertiary hospital and not a dedicated cancer center, it is policy to treat the patient presenting with fevers whilst undergoing chemotherapy with piperacillin/tazobactam and gentamicin initially, and indeed, to assume all patients are neutropenic until proven otherwise, particularly when seen in the emergency department. As described, a proportion of these patients are not neutropenic (28.5%) and in these instances antibiotics are often rapidly de-escalated or ceased, depending upon the clinical circumstances. This leads to a degree of ‘over-treatment’ however also represents a safe and conservative management strategy for our highest risk patients.

Of interest, we observed no carbapenem resistance in our cohort, though this is observed within our region. Carbapenem-resistant *Pseudomonas aeruginosa* represent approximately 5% of isolates state-wide, and a small number (< 1%) of bloodstream isolates of *Enterobacteriaceae* nationally are carbapenem-resistant [25]. At a hospital-wide level, ESBL producing isolates accounted for 2.4–9.2% of *E coli* BSI and 3.9–8.0% of *K*. *pneumoniae* BSI annually, during the study period; gentamicin resistance was present in 46.9% of these isolates, in keeping with our reported rate. Interestingly, significant rates of MRSA bacteremia were seen in the hospital-wide group (10–22% of *S*. *aureus* isolates), but not observed within the hematology patients studied. This low resistance rate in our population likely reflects low carriage of resistant organisms in the community, in combination with generally low rates of carbapenem and vancomycin use in our patient cohort, in addition to conservative prescribing of fluoroquinolones as prophylaxis. A meta-analysis assessing the role of fluoroquinolone prophylaxis published in 2005, which examined studies performed in the 25 years prior suggested a benefit in all-cause and infectious mortality with use of these drugs compared to placebo, but this was traded for significant increases in downstream resistance to empiric antibiotic therapies [26]. Of note, the rates of infectious mortality varied considerably across the individual trials included, with infectious mortality in the placebo group of up to 27.2%, compared with rates of up to 22.8% in the fluoroquinolone arm. A single large RCT, specifically assessing the high-risk hematology patient group, demonstrated fewer fevers with the use of levofloxacin prophylaxis, but without differences in infectious mortality or proven BSI [27]. The downstream consequences of widespread fluoroquinolone prophylaxis are now becoming evident with some centers reporting near-universal fluoroquinolone-resistant isolates from urine and blood of hematology patients [28]. Our low mortality highlights the safety of a fluoroquinolone prophylaxis-free approach in a setting where first line therapy is microbiologically inappropriate in 11.6% of patients.

We observed limited utility in the routine repetition of blood culture collection for further fevers unless the recurrent febrile episode was associated with hemodynamic instability or other concerning clinical deterioration. These data, therefore, support a conservative reculture practice, and use of clinical deterioration as a driver for repeat culture collection, rather than fever in isolation.

We acknowledge the limitations of the data presented, as the study was retrospective, and conducted within a single center. Though CVLs are universally employed (most commonly peripherally inserted central catheters) documentation regarding blood culture site was poor, and thus we could not reliably ascertain whether culture results were from a central line or peripheral venipuncture. We have elected to exclude CoNS (and other organisms determined to represent contamination) from our reported rates of blood culture positivity, because in our local patient group (who are not allogeneic or autologous transplant recipients) they are considered contaminants and broadly accepted to be of low pathogenicity [16]. Thus, we are cautious in comparing our rates of culture positivity to that reported in other centers where CoNS are routinely included. Additionally, the low mortality rate precludes meaningful statistical analysis of risk factors for mortality, and the retrospective nature of the study means that other useful parameters (e.g. MASCC index [29, 30]) could not be included. We have chosen to analyze the cumulative BC and resultant BSI in our patient group over the five year period because the resulting data has practical application for the ongoing care of our patients, compared with an analysis using person-time as a denominator, which is an alternative approach used in studies of this type.

## Conclusion

In summary, we demonstrate that BSI-related mortality is low in our center, gram-negative organisms are isolated most commonly, and resistance rates are low overall. The data supports our current practices and provides valuable insights into our patient outcomes, however, re-culture in the context of recurrent fever without further clinical deterioration appears to be of little utility. The key difference between our center and other recent international reports is the low rate of resistant organisms, and we highlight the safety of a fluoroquinolone prophylaxis-free approach in terms of infectious mortality in our setting. Currently, blood culture is required for the diagnosis of BSI and the identification of resistance in almost all clinical settings, but new molecular methods continue to be developed and these may prove to be beneficial in the investigation of febrile neutropenic patients, as a small but significant rate of resistance persists across isolates amongst this high-risk cohort.
